# Analysis of the efficacy of avatrombopag for the delayed platelet engraftment after allogeneic hematopoietic stem cell transplantation for aplastic anemia

**DOI:** 10.3389/fmed.2025.1626325

**Published:** 2025-08-08

**Authors:** Xinhe Zhang, Jia Feng, Zhengwei Tan, Herui Zhang, Huijin Hu, Yuechao Zhao, Dijiong Wu, Yu Zhang, Liqiang Wu, Tonglin Hu, Zhengsong Yan, Baodong Ye, Wenbin Liu

**Affiliations:** ^1^The First Affiliated Hospital of Zhejiang Chinese Medical University (Zhejiang Provincial Hospital of Chinese Medicine), Hangzhou, China; ^2^Nanyang First People’s Hospital, Nanyang, China; ^3^The First Clinical College of Zhejiang Chinese Medical University, Hangzhou, China

**Keywords:** DPE, avatrombopag, Allo-HSCT, TPO-RA, RhTPO

## Abstract

**Background:**

Delayed platelet engraftment (DPE) after allo-HSCT lacks standard therapy. Avatrombopag (AVA), a second-generation TPO agonist, is often delayed until transfusion-related events occur, potentially harming high-risk recipients.

**Objectives:**

We compared recombinant human thrombopoietin (rh-TPO) with early AVA switching for treating DPE in aplastic anemia (AA) patients post-allo-HSCT to optimize management strategies.

**Methods:**

This single-center study retrospectively enrolled 154 consecutive AA patients receiving allo-HSCT at Zhejiang Provincial Hospital of Traditional Chinese Medicine (March 2019–September 2023). Of these, 39 deemed high-risk for poor platelet engraftment (advanced donor/recipient age, low CD34 + dose, etc.) were non-randomly assigned: (1) AVA group (*n* = 11), switched to avatrombopag if platelets remained <30 × 10^9^/L on day +14 or <50 × 10^9^/L on day +21; (2) rh-TPO group (*n* = 28), continued rh-TPO monotherapy. Allocation followed clinician judgment and patient consent.

**Results:**

Our findings revealed that the 1-year overall survival (OS) rate was notably higher in AVA group (100% vs. 78.6%, *p* = 0.106). And the complete remission (CR) rate in the AVA group was significantly higher than that in the rh-TPO group at 3 and 6 months after transplantation(100% vs. 67.9%, *p* = 0.032; 100% vs. 71.4%, *p* = 0.047). At 3 months post transplantation, the platelet engraftment rate in the AVA group was significantly higher than that in the rhTPO group (67.9% vs. 100%, *p* = 0.04). The median time to achieve platelet engraftment was 20 (13, 25) days for the AVA group and 23 (10, 68) days for the rh-TPO group. Additionally, the AVA group reached platelet counts of 30, 50, and 125 × 109/L more rapidly than the rh-TPO group. Furthermore, at 3 months post-transplantation, the median platelet transfusion volume of AVA group was less than rh-TPO group (63 U vs. 82 U, *p* = 0.141).

**Conclusion:**

For patients identified as being at high risk for poor platelet engraftment following allo-HSCT, early transition to AVA can significantly reduce the duration of DPE and promote platelet recovery post-transplantation. This strategy has the potential to enhance patient survival rates and overall outcomes.

## Introduction

Aplastic anemia (AA) is a prevalent clinical condition distinguished by bone marrow failure, with its primary clinical presentations being anemia, bleeding, and infections (1–3). Allogeneic hematopoietic stem cell transplantation (allo-HSCT) is recognized as a curative treatment for AA, substantially enhancing the survival rates among AA patients (4, 5). However, allogeneic hematopoietic stem cell transplantation (allo-HSCT) is often accompanied by various complications as the same time. These include graft-versus-host disease (GVHD), delayed platelet engraftment (DPE), and poor graft function (PGF), which severely impact patients’ quality of life and survival rates (6). In recent years, the incorporation of post-transplant cyclophosphamide (PT-CY) into haploidentical hematopoietic stem cell transplantation has effectively reduced the incidence of post-transplant GVHD. Nevertheless, the incidence of DPE remains high at 5–37%, which can result in adverse clinical outcomes such as bleeding, secondary infection, and increased transplant-related mortality (7–10).

Engraftment is the key indicator of the success of allo-HSCT, with platelet engraftment being particularly crucial. Platelet engraftment is characterized by the first occurrence of three consecutive days where the platelet count reaches 20,000/ml or more, without the need for platelet transfusions for the 7 days prior (10–12). Post allo-HSCT platelet engraftment varies among patients, occurring from 2 weeks to several months post-transplant (13). Delayed platelet engraftment (DPE), with a 9 to 40% clinical incidence, is a severe allo-HSCT complication that increases transplant-related mortality (TRM) and impacts prognosis (9, 14). Its exact etiology is unclear, but factors like donor-recipient age, graft source, conditioning regimen intensity, donor CD34 + cell count, Cytomegalovirus (CMV)/Epstein–Barr virus(EBV) infections, and GVHD can lead to DPE by inhibiting bone marrow megakaryocyte differentiation or damaging stroma function (15, 16). All the aforementioned factors constitute high-risk factors for impaired platelet engraftment post-transplantation. Our prior studies also indicate that around day 30 post-transplant is a high-risk period for viral reactivation and acute GVHD, which can trigger DPE, complicating the clinical scenario and endangering patient survival and quality of life (17, 18). Thus, early platelet engraftment, especially achieving a higher count before day 30, can reduce bleeding risk, mitigate transplant complications, and enhance prognosis.

Research has established that TPO/thrombopoietin peceptor agonists (TPO-RAs) bind to the TPO receptor (TPO-r), thereby promoting platelet production through mechanisms that reduce megakaryocyte apoptosis and enhance their maturation (19, 20). Moreover, TPO-RAs are capable of overcoming interferon-*γ* (IFN-γ) signaling pathway blockades, thus promoting the survival and proliferation of hematopoietic stem and progenitor cells (21). Avatrombopag, a second-generation oral TPO-RA, precisely targets the c-MPL receptor on megakaryocytes, activating the JAK–STAT signaling pathway. This activation stimulates megakaryocyte proliferation and differentiation, ultimately leading to an increase in platelet production. Having received FDA approval, avatrombopag is widely utilized in the treatment of immune thrombocytopenia and severe aplastic anemia (22, 23). Recent studies further confirm its efficacy in promoting post-transplant platelet engraftment and decreasing platelet transfusions in DPE patients (24, 25). Nevertheless, there is a notable absence of clear guidelines regarding the optimal timing and specific efficacy of AVA for DPE. The majority of clinical studies concentrate on treating patients diagnosed with platelet engraftment failure (≥ + 30 days) rather than implementing early intervention prior to this diagnosis. This approach may result in missed optimal treatment windows, prolonged severe thrombocytopenia, and heightened risks of serious bleeding, particularly for high-risk patients.

Therefore, we conducted this study to compare the effects of post-transplant rh-TPO monotherapy and early AVA switch on platelet engraftment and prognosis in AA patients with high-risk platelet engraftment failure.

## Cases and methods

### Case data

This study enrolled 154 patients with AA who underwent allo-HSCT at the First Affiliated Hospital of Zhejiang Chinese Medical University (Zhejiang Hospital of Traditional Chinese Medicine) from March 1, 2019 to September 30, 2023. Of these, 39 patients had high-risk factors for platelet engraftment failure. The patients were divided into two groups based on clinical response: Patients failing to achieve platelet count ≥30 × 10^9^/L on day +14 or ≥ 50 × 10^9^/L on day +21 were switched to AVA (AVA group, *n* = 11) per physician discretion, while responders continued rh-TPO monotherapy (rh-TPO group, *n* = 28). All participants were selected based on the following criteria:

1. Inclusion criteria (fulfillment of all major criteria plus at least one minor criterion):

Major criteria (all are mandatory): A. Age between 15 and 70 years, inclusive, irrespective of gender; B. ECOG performance status score of 0–3; C. A definitive diagnosis of aplastic anemia, with exclusion of other hematological disorders; D. Undergone allogeneic hematopoietic stem cell transplantation; E. Demonstrated adequate comprehension and voluntarily signed an informed consent form.

Minor criteria (at least one must be satisfied): Minor criteria (at least one must be met): A. Donor age is over 50 years or the donor’s peripheral CD34 + stem cell count is less than 10/μL (26, 27). B. The infused CD34 + stem cell count is less than 3.0 × ×10^6^/kg (28–30). C. Donor-specific antibodies (DSA) have a mean fluorescence intensity (MFI) greater than 10,000 (31, 32). D. TPO was administered to facilitate platelet engraftment on day +4 post-transplant, yet the platelet count remained below 30 × ×10^9^/L on day +14 or below 50 × 10^9^/L on day +21.

1. Exclusion criteria: A. A history of other malignancies; B. Patients who did not undergo allogeneic hematopoietic stem cell transplantation; C. Patients who did not receive TPO treatment post-transplant; D. Uncontrolled active infections; E. HIV infection, uncontrolled hepatitis B (HBV), hepatitis C (HCV), or syphilis infection; F. Lack of autonomy or unwillingness to sign the informed consent form.

### Pre-treatment regimen and GVHD prevention protocol

#### Pre-treatment regimen

All 39 AA patients in this study underwent allo-HSCT and received different conditioning regimens based on their baseline conditions and donor types. Specifically: Seven patients received a modified Bu/Cy (Busulfan + Cyclophosphamide) regimen, comprising intravenous busulfan (Bu) 3.2 mg/kg/day on -7d to -6d, fludarabine (Flu) 30 mg/m^2^/day on -10d to -7d, cyclophosphamide (CTX) 50 mg/kg/day on -5d to -2d, and antithymocyte globulin (ATG) 2.5 mg/kg/day on -5d to -2d. Twenty-one patients received the FCA (Fludarabine + Cyclophosphamide + ATG) regimen, which included intravenous fludarabine (Flu) 30 mg/m^2^/day on -9d to -5d, cyclophosphamide (CTX) 20–40 mg/kg/day on -5d to -2d, and antithymocyte globulin (ATG) 2.5 mg/kg/day on -5d to -2d. Eleven patients received the FABT (fludarabine + ATG + busulfan + thiotepa) regimen, consisting of intravenous busulfan (Bu) 0.8 mg/kg/day -4d, fludarabine (Flu) 30 mg/m^2^/day on -7d to -3d, cyclophosphamide (CTX) 25 mg/kg/day on -4d to -2d, thiotepa (TT) 5 mg/kg/day on -4d, and antithymocyte globulin (ATG) 2 mg/kg/day on -7d to -5d.

#### GVHD prevention protocol

Patients conditioned with modified Bu/Cy or FCA received GVHD prophylaxis comprising a calcineurin inhibitor (cyclosporine or tacrolimus), short-course methotrexate, and mycophenolate mofetil (MMF). In contrast, the FABT protocol substituted reduced-dose antithymocyte globulin and post-transplant cyclophosphamide for methotrexate, while retaining a calcineurin inhibitor and MMF. All calcineurin inhibitors were tapered off and discontinued no later than 1 year after transplantation. The specific GVHD drug regimens, diagnostic criteria, and grading all followed the latest expert consensus (33).

### Management of DSA positivity

Prior to stem-cell infusion, all patients were tested for donor-specific antibodies (DSA). Patients with DSA levels ≥5,000 underwent plasma exchange on -11d and received intravenous rituximab at 375 mg/m^2^on -10d and -3d to deplete antibodies.

### Engraftment criteria

#### Neutrophil engraftment

The first day with a sustained absolute neutrophil count (ANC) of at least 0.5 × 10^9^/L for 3 consecutive days. Platelet engraftment: The first day with a sustained platelet count of at least 20 × 10^9^/L (after cessation of platelet transfusions) for 7 consecutive days (11, 12).

### Treatment

In this study, all patients received rh-TPO at 300 U/kg/day and G-CSF at 5–10 μg/kg/day starting on +4d after allo-HSCT to promote platelet and granulocyte engraftment. The rh-TPO group included 28 patients who did not receive avatrombopag or other TPO-RAs. The AVA group included 11 patients who were switched to avatrombopag treatment (at a dose of 40 mg orally once daily) when peripheral blood platelet counts remained below 30 × 10^9^/L after 10 to 14 days of rh-TPO treatment. Treatment was discontinued in both groups when platelet counts exceeded 80 × 10^9^/L or when severe adverse reactions occurred. The study was approved by the Ethics Committee of The First Affiliated Hospital of Zhejiang Chinese Medical University, and all consent forms approved by the institution were signed.

### Response determination criteria

The complete response (CR) was defined as the platelet ≥50 × 10^9^ /L for at least 7 consecutive days without transfusion. Partial response (PR) was defined as PLT of (20–50) × 10^9^/L without PLT transfusion for 7 continuous days (25). Adverse events and severe adverse events were evaluated according to the National Cancer Institute Common Terminology Criteria for Adverse Events (NCI-CTCAE) version 5.0.

Platelet-engraftment milestones were defined as the first day of three consecutive days with platelet counts ≥30, ≥50 and ≥125 × 10^9^/L without transfusion support. These thresholds correspond, respectively, to a clinically meaningful reduction in bleeding risk (≥30 × 10^9^/L), the safe discontinuation of prophylactic platelet transfusions (≥50 × 10^9^/L), and full restoration of the normal range (≥125 × 10^9^/L) (34–36).

### Statistical analyses

Two-tailed t-tests or Kruskal-Wallis tests were utilized to assess differences in continuous variables among groups. For categorical variables, comparisons were made using the chi-squared (*χ*^2^) test or Fisher’s exact test, as appropriate. Univariate and multivariate logistic regression analyses were performed to identify risk factors associated with CR. Variables from the univariate analysis with a *p*-value of less than 0.05 or those with a significant correlation were advanced to the multivariate analysis. The overall survival (OS) probability was estimated using the Kaplan–Meier method and differences were assessed with the log-rank test. Statistical significance was set at a *p*-value of less than 0.05. All data analyses were conducted using SPSS version 25.0.

## Results

### Patient general characteristics

This study enrolled 39 patients with AA who underwent allo-HSCT, comprising 25 males (64.1%) and 14 females (35.9%) with a median age of 36.3 ± 12 years. Baseline characteristics were well balanced between the two groups, indicating comparable populations. The rh-TPO group consisted of 28 patients, 10 (35.7%) of whom developed aGVHD (5 cases of grade I aGVHD and 5 cases of grade II-IV aGVHD) at a median of 39.55 ± 33.56 days post-transplant. Additionally, 9 patients (32.1%) in this group developed cGVHD at a median of 169.33 ± 24.19 days. The AVA group included 11 patients, with no aGVHD occurrences and 1 case (9.1%) of cGVHD. Among them, only two patients received ruxolitinib for GVHD, using a reduced-dose regimen of 5 mg twice daily at our center. EBV reactivation rates post-transplant were 60.7% in the rh-TPO group and 45.5% in the AVA group, with no significant difference (*p* = 0.393). CMV reactivation rates were 50% in the rh-TPO group and 27.3% in AVA group, also without significant difference (*p* = 0.19).

### Hematopoietic reconstruction

At 3 months post-transplantation, the platelet engraftment rate was significantly higher in the AVA group compared to the rh-TPO group (100% vs. 67.9%, *p* = 0.04). The median time to platelet engraftment was 20 (13, 25) days in the AVA group, compared with 21 (17, 27) days in the rh-TPO group (*p* = 0.49). At 1 month post-transplantation revealed a significantly higher megakaryocyte count in the AVA group than in the rh-TPO group (*p* = 0.045). The median time to achieve platelet counts of 20 × 10^9^/L and 50 × 10^9^/L of AVA group were earlier than rh-TPO group (25d vs. 27d, 29d vs. 37d). By 3 months post-transplantation, the median number of platelet transfusions in the AVA group was 63 (44.5, 83.5) units, which was lower than the 82 (54.5, 101) units in rh-TPO group (*p* = 0.141). The mean hemoglobin (Hb) level in the AVA group was higher than that in the rh-TPO group during months 1–5 post-transplantation (*p* > 0.05).

### Follow-up

At the 100-day mark post-transplant, the CR rate in the converted group reached 100%, whereas the rh-TPO group had a PR rate of 7.1% and a CR rate of 67.9%. By 180 days post-transplant, the CR rate in the converted group was sustained at 100%, compared to the rh-TPO group’s PR rate of 3.5% and a CR rate of 71.4%. Tsotentially impacting the CR rate, including patient age, gender, and the timing of avatrombopag initiation, were analyzed using both univariate and multivariate approaches. The findings suggested that early initiation of avatrombopag treatment significantly improved the CR rate in patients (*p* = 0.014 and *p* = 0.039).

Three months post-transplantation, the AVA group achieved a complete remission (CR) rate of 100%, while the rh-TPO group had a partial remission (PR) rate of 10.7% and a CR rate of 67.9%. At 6 month mark, the CR rate in the AVA group remained at 100%, compared to a PR rate of 7.1% and a CR rate of 71.4% in the rh-TPO group. The CR rate in the AVA group was significantly higher than that in the rh-TPO group at both 3 months (100% vs. 67.9%, *p* = 0.032) and 6 months (100% vs. 71.4%, *p* = 0.047). The 1-year overall survival (OS) rate was higher in the AVA group than in the rh-TPO group (100% vs. 78.6%, *p* = 0.106). Univariate regression analysis was conducted with factors such as patient age, gender, and early switch to AVA treatment that might affect the CR rate. Variables with statistical significance (*p* < 0.05) or clinical relevance were included in the multivariate regression analysis. Both analyses indicated that early switch to AVA treatment could improve the CR rate (*p* = 0.014, *p* = 0.039).

### Complications

No severe adverse event was reported in our study. It indicated that rh-TPO and AVA played a relatively safe role in this study.

## Discussion

DPE is a life-threatening complication following allogeneic hematopoietic stem cell transplantation, potentially increasing transplant-related mortality (TRM). Factors such as the presence of anti-HLA antibodies, advanced age of donors or recipients, and low pre-conditioning platelet counts can impact platelet engraftment, leading to post-transplant thrombocytopenia, which affects patient survival and prognosis (15, 26). Currently, many clinical centers employ rhTPO to facilitate platelet engraftment post-transplantation, with generally good clinical outcomes. However, its efficacy can be variable, particularly in patients with high-risk factors (15). Avatrombopag, a second-generation thrombopoietin (TPO) receptor agonist, has been effective in treating chronic immune thrombocytopenia, offering earlier and more sustained responses (22, 23). In recent years, avatrombopag has also been utilized for DPE post-transplant (37). Yet, in clinical practice, it is often administered only after the diagnosis of post-transplant thrombocytopenia, resulting in treatment delays and a less favorable prognosis for some patients (15, 16, 27). In this study, we selected 11 AA patients with high-risk factors for platelet engraftment failure. These patients were switched to AVA for preemptive treatment early after transplantation due to poor response to rh-TPO therapy. We compared their platelet engraftment with that of 28 patients who received only rh-TPO therapy after transplantation. The results showed that at 3 and 6 months post-transplantation, the CR rate was higher in the AVA group than in the rh-TPO group (100% vs. 67.9%, *p* = 0.032; 100% vs. 71.4%, *p* = 0.047). Both univariate analysis and multivariate regression analysis indicated that an early switch to AVA treatment could improve the CR rate (*p* = 0.014, *p* = 0.039).

AVA and eltrombopag bind to the same site within the transmembrane domain of the thrombopoietin receptor (TPO-R), thereby enhancing platelet production through the stimulation of megakaryocyte proliferation and differentiation in the bone marrow. This binding site is notably distinct from the region where thrombopoietin (TPO) binds (19). Upon TPO binding to the transmembrane domain of TPO-R, it triggers downstream signaling pathways, including STAT3/5, AKT, and ERK (38, 39). In contrast, eltrombopag robustly activates all downstream pathways of TPO-R, exhibiting a more potent effect on promoting megakaryocyte proliferation and platelet production, a property shared by AVA (40). A retrospective study has shown that eltrombopag effectively mitigates poor graft function after hematopoietic cell transplantation. It does so by directly stimulating megakaryocyte proliferation and platelet production through activation of the TPO receptor, c-Mpl, and by coaxing quiescent hematopoietic stem and progenitor cells (HSPCs) back into cycle, thereby reconstituting hematopoietic capacity (41). However, unlike eltrombopag, AVA lacks the potential for cation or iron chelation and does not impose dietary restrictions during its administration (19). A retrospective study, in which AVA was administered to patients with persistent immune thrombocytopenia (PIT) and secondary immune thrombocytopenia (SFPR), demonstrated a complete response (CR) rate of 81.3% (13 out of 16 patients) (42). Furthermore, an experimental study involving the transplantation of human fetal liver CD34 + cells into mice confirmed that AVA more significantly increases platelet counts compared to eltrombopag (43). An *in vitro* cellular study has confirmed that the concurrent administration of rh-TPO and AVA to human CD34 + selected stem cells yields a synergistic effect, attributed to their distinct binding sites (19). These findings are closely related to the faster platelet engraftment observed in AA patients who switched to AVA treatment in our study. At 1-month post-transplantation, we noted a significant difference in megakaryocyte counts between the two groups (*p* = 0.045). The AVA group had a mean of 12 (3,29) megakaryocytes in the bone marrow, compared to just 3 (0,8) in the rh-TPO group. This underscores AVA is superior efficacy in promoting megakaryocyte differentiation and maturation over rh-TPO. The platelet engraftment rate was significantly higher in the AVA group than in the rh-TPO group (100% vs. 67.9%, *p* = 0.04), with engraftment occurring earlier in the AVA group (20 days vs. 21 days). The AVA group also reached platelet counts of 20 × 10^9^/L and 50 × 10^9^/L sooner than the rh-TPO group (*p* = 0.179, *p* = 0.041). Moreover, by 3 months post-transplantation, the AVA group required fewer platelet transfusions (63 units vs. 82 units, *p* = 0.141). Thus, switching to AVA treatment early post-transplantation can hasten platelet engraftment and boost engraftment rates. This holds significant promise for enhancing the survival and prognosis of high-risk patients post-transplantation.

T cell-mediated immune responses generate interferon gamma (IFN-*γ*), which interferes with the TPO/TPO-R (c-Mpl) interactions and contributes to the destruction of hematopoietic stem and progenitor cells (HSPCs) by suppressing downstream TPO signaling. This results in a significantly reduced HSPC count in severe aplastic anemia (SAA) patients. Eltrombopag, however, does not interact with IFN-*γ*, enabling it to evade the inhibitory impact of IFN-γ on the TPO signaling pathway. Instead, it directly targets the transmembrane domain to foster HSPC survival and proliferation (21). This mechanism may also apply to AVA (19). Zoe McQuilten and colleagues have proposed that AVA binding to the TPO receptor (c-Mpl) on hematopoietic stem cells activates intracellular signaling pathways critical for the production of megakaryocytes, platelets, hemoglobin, and neutrophils (20). Yongsheng Ruan and colleagues conducted a study involving 30 patients, which indicated that the administration of AVA to post-transplant patients not only enhances platelet counts but also elevates hemoglobin levels and certain neutrophil counts (37). Echoing the findings of the aforementioned experiment, in our current research, while there were no statistically significant differences in hemoglobin levels between the two groups post-transplantation—potentially attributable to the modest sample size of our study—it is observable from the accompanying figure that the average hemoglobin levels in the converted group consistently surpassed those in the rh-TPO group on a monthly basis. However, the disparities in myeloid engraftment rates and engraftment timelines did not reach statistical significance.

However, this study also has some limitations. There was a statistically difference in the incidence of aGVHD between the two groups after transplantation, which may have a potential impact on the results. Additionally, the study cohort was small, and the follow-up period for both groups was relatively short. Therefore, larger-scale prospective randomized controlled trials are needed in the future to further validate these findings.

### Summary

In conclusion, for AA patients with high-risk factors for poor platelet engraftment undergoing allo-HSCT, an early switch to AVA treatment can enhance platelet engraftment post-transplantation, lower the incidence of DPE, and significantly boost overall survival and prognosis.

## Data Availability

The datasets presented in this study can be found in online repositories. The names of the repository/repositories and accession number(s) can be found in the article/Supplementary material.
