# Correction: Analysis of the efficacy of avatrombopag for the delayed platelet engraftment after allogeneic hematopoietic stem cell transplantation for aplastic anemia

**DOI:** 10.3389/fmed.2025.1696198

**Published:** 2025-09-24

**Authors:** Xinhe Zhang, Jia Feng, Zhengwei Tan, Herui Zhang, Huijin Hu, Yuechao Zhao, Dijiong Wu, Yu Zhang, Liqiang Wu, Tonglin Hu, Zhengsong Yan, Baodong Ye, Wenbin Liu

**Affiliations:** ^1^The First Affiliated Hospital of Zhejiang Chinese Medical University (Zhejiang Provincial Hospital of Chinese Medicine), Hangzhou, China; ^2^Nanyang First People's Hospital, Nanyang, China; ^3^The First Clinical College of Zhejiang Chinese Medical University, Hangzhou, China

**Keywords:** DPE, avatrombopag, Allo-HSCT, TPO-RA, RhTPO

Affiliation “The First Affiliated Hospital of Zhejiang Chinese Medical University, Hangzhou, China” was erroneously listed as the third affiliation, it should be listed as the first affiliation and edited to “The First Affiliated Hospital of Zhejiang Chinese Medical University (Zhejiang Provincial Hospital of Chinese Medicine), Hangzhou, China”.

Affiliation “Nanyang First People's Hospital, Nanyang, China” was erroneously listed as the first affiliation, it should be listed as the second affiliation.

Affiliation “The First Clinical College of Zhejiang Chinese Medical University, Hangzhou, China” was erroneously listed as the second affiliation, it should be listed as the third affiliation.

Authors Xinhe Zhang, Jia Feng and Zhengwei Tan should be listed as equal contributing authors. “These authors contributed equally to this work” has been added as a footnote.

The original version of this article has been updated.

