# Supplementary material for: Analysis of the efficacy of avatrombopag for the delayed platelet engraftment after allogeneic hematopoietic stem cell transplantation for aplastic anemia
Source: Front Med (Lausanne). 2025 Aug 8;12:1626325. doi: 10.3389/fmed.2025.1626325 (PMC12370753; doi:10.3389/fmed.2025.1626325)
Supplement: Supplementary file 1 [file Data_Sheet_1.docx]

|  |  |
| --- | --- |
| Figure 1: Platelet Engraftment Rates in  Two Groups Post-Transplantation | Figure 2: Platelet Counts in Two Groups  Post-Transplantation |
|  |  |
| Figure 3: Average Hemoglobin Levels in  Two Groups Post-Transplantation | Figure 4: Complete Response (CR) and Partial  Response (PR) Rates in Two Groups  at +6 Months After Transplantation |

|  |  |
| --- | --- |
| Figure 5: 1-Year Overall Survival (OS) Rates Between Two Patient Groups Following Transplantation |  |
|  |  |
| Figure 6: Time to Platelet Engraftment  Post-Transplantation | Figure 7: Time to Achieve a Platelet Count of 20×10⁹/L Post-Transplantation |
|  |  |
| Figure 8: Time to Achieve a Platelet Count of  50×10⁹/L Post-Transplantation | Figure 9: Cumulative Platelet Transfusion Volume at +3 Months Post-Transplantation |

Table 1 Characteristics of patients

| Characteristic | All | AVA group | Rh-TPO group | P Value |
| --- | --- | --- | --- | --- |
|  | N=39 | N=11 | N=28 |  |
| Age at transplantation, Mean±SD | 36.3±13.2 | 34.1±20.1 | 37.2±9.7 | 0.634 |
| Sex, n(%) |  |  |  | 0.478 |
| Male | 25（64.1） | 6（54.5） | 19（67.9） |  |
| Female | 14（35.9） | 5（45.5） | 9（32.1） |  |
| Donor type, n (%) |  |  |  | ＞0.999 |
| HLA-matched related | 4（10.3） | 1（9.1） | 3（10.7） |  |
| HLA-mismatched related | 35（89.7） | 10（90.9） | 25（89.3） |  |
| Basal platelet count, M(P_25_，P_75_) | 11（7,14） | 13（7,16） | 10.5(7,13.8) | 0.471 |
| ABO matching, n(%) |  |  |  | 0.48 |
| Matched | 19(48.7) | 4(36.4) | 15(53.6) |  |
| Mismatched | 20(51.3) | 7(63.6) | 13(46.4) |  |
| CMV infection, n (%) |  |  |  | 0.19 |
| No | 22(56.4) | 8（72.7） | 14（50） |  |
| Yes | 17(43.6) | 3（27.3） | 14（50） |  |
| EBV infection, n (%) |  |  |  | 0.393 |
| No | 17(43.6) | 6（54.5） | 11（39.3） |  |
| Yes | 22(56.4) | 5（45.5） | 17（60.7） |  |
| Donor-patient sex matching, n (%) |  |  |  | 0.82 |
| Female to male | 8（20.5） | 2（18.2） | 6（21.4） |  |
| Others | 31（79.5） | 9（81.8） | 22（78.6） |  |
| Megakaryocyte count, M(P_25_，P_75_)  (+1 month) | 3.5（0,17.8） | 12（3,29） | 3（0,8） | 0.045 |
| Plate-producing megakaryocyte count, M(P_25_，P_75_) (+1 month) | 0（0,2） | 1（0,3） | 0（0,1.5） | 0.129 |
| aGVHD |  |  |  | 0.037 |
| Yes | 10(25.6) | 0 | 10(35.7) |  |
| No | 29(74.4) | 11(100) | 18(64.3) |  |
| cGVHD |  |  |  | 0.228 |
| Yes | 10(25.6) | 1(9.1) | 9(32.1) |  |
| No | 29(74.4) | 10(90.9) | 19(67.9) |  |

Table 2 Analysis of Risk Factors Related to CR in Patients

| Factors | Univariate analysis | | Multivariate analysis | |
| --- | --- | --- | --- | --- |
|  | OR（95% CI） | P Value | OR（95% CI） | P Value |
| Age | 0.971(0.923,1.021) | 0.250 | - | - |
| Sex | 1.950(0.508,7.489) | 0.331 | - | - |
| HLA | 0.353(0.033,3.731) | 0.387 | 0.102(0.005,2.144) | 0.142 |
| Basal platelet count | 1.108(0.950,1.292) | 0.192 | 1.147(0.932,1.412) | 0.196 |
| Megakaryocyte count (+1 month) | 1.033(0.975,1.096) | 0.273 | - | - |
| Plate-producing megakaryocyte count (+1 month) | 1.033(0.922,1.157) | 0.581 | - | - |
| PLT implantation or not | 6.045(1.063,34.37) | 0.042 | 2.720(0.379,19.68) | 0.322 |
| Converted | 15.45(1.728,138.2) | 0.014 | 12.72(1.136,142.5) | 0.039 |
| CMV infection | 0.400(0.109,1.466) | 0.167 | 0.231(0.039,1.349) | 0.104 |
| EBV infection | 0.700(0.195,2.511) | 0.584 | - | - |
| Donor-patient sex | 0.640(0.130,3.155) | 0.583 | - | - |

*sex :0 male 1female；HLA :0 matched 1 mismatched；PLT implantation or not: 0 no 1 yes；converted or not: 0 no 1 yes.
